# What Do We Know About Young Adult Cardiac Patients' Experience? A Systematic Review

**DOI:** 10.3389/fpsyg.2020.01119

**Published:** 2020-07-07

**Authors:** Jonathan Journiac, Christel Vioulac, Anne Jacob, Coline Escarnot, Aurélie Untas

**Affiliations:** Université de Paris, LPPS, Boulogne-Billancourt, France

**Keywords:** young, adults, cardiac, experience, review

## Abstract

**Background:** Studies interested in patients coping with a cardiac illness usually focus on children, teenagers, and adults above the age of 55. Apart from the field of congenital heart diseases, there is a general lack of literature regarding young adult cardiac patients (18–55 years old) who seem to cope with psychosocial issues. Therefore, the objective of this paper was to gather all the research carried out concerning the psychological experiences of young adult cardiac patients.

**Methods and Results:** A comprehensive, systematic review was conducted on quantitative, qualitative, and mixed-method studies in PsycINFO, PubMed, ScienceDirect, and Cochrane Library databases. Out of the 10,747 articles found, 32 were included. While we aimed to include many cardiac diseases, coronary patients dominated the data. Five main themes emerged: emotional states (depression, anxiety, emotional distress, and stress), quality of life (health-related quality of life, physical functioning, and sexuality), adjusting to the medical environment (coping with the disease, health behavior change, financial barriers, and interactions with medical professionals), social life (social support and work), and identity (parenthood, new challenges, and new meanings). The results highlighted that their levels of depression, anxiety, stress, and quality of life were sometimes worse than in the general population and than in older and younger patients coping with a cardiac illness. Social isolation, identity changes, work, and parenthood were the specific challenges that this population had to face. Furthermore, young adult cardiac patients showed worse health behavior profiles than the general population and felt that they lacked information from professionals, especially regarding sexuality. Compared to men, women had worse psychosocial outcomes, especially regarding depression, stress, emotional distress, and quality of life.

**Conclusions:** Young adult cardiac patients are to be considered with their own identity and challenges. They may be in need of specific interventions, some dedicated to women, and better communication is necessary with their families and professional caregivers so as to improve the patient's mental health, quality of life, coping skills, and adherence.

## Introduction

Cardiac illnesses, such as myocardial infarctions (MI), coronary diseases, and cardiac arrests are most common among adults over 55 years old, yet since congenital heart defects can be discovered as early as during childhood, young people might also find themselves coping with a cardiac illness (HAS, 2016; Al-Dury et al., 2017; Inserm, 2019).

Young adult patients represent 10–23% of all men and women with a coronary illness (Rosengren et al., 2006; Bangalore et al., 2012; Izadnegahdar et al., 2014). In cardiac settings, adults are classified as young when they are between 18 and 40 years old (McDonough, 2009). However, the age limit of young men with coronary illness is often considered by cardiologists to be 45 and, for women, who are protected by their hormones, it could rise up to 55 as their symptoms and peak illness incidence happen 10 years later (Rosengren, 2001; Estève et al., 2008; Vazquez et al., 2008; Schiele and Chopard, 2014).

Being younger is associated with more adverse psychological outcomes in cardiac settings: young adult patients tend to have more difficulties in adjusting to their disease, present unique challenges and coping behaviors, and experience more depression, anxiety, post-traumatic stress symptoms, and decreased quality of life (QoL) compared to older patients (Sears et al., 1999, 2009; Friedmann et al., 2006; Mallik et al., 2006; Vazquez et al., 2008; McDonough, 2009; Larimer et al., 2016; Andonian et al., 2018; Richardson et al., 2018). Additionally, there is growing evidence suggesting that young cardiac women are a subgroup especially at risk for mortality and psychological difficulties (Mallik et al., 2006; Vazquez et al., 2008; Sears et al., 2009; Pelletier et al., 2016b; Sabbag et al., 2017; Vaccarino et al., 2019).

However, most studies include samples whose mean age is above 55 years old and rarely focus on younger adult patients (Whalley et al., 2014; Protogerou et al., 2015; Easton et al., 2016; Ooi et al., 2016; Craner et al., 2017). Studies that include young adults coping with a cardiac illness are scarce (McDonough, 2009; Wong et al., 2017). Systematic reviews interested in the psychosocial aspects of individuals coping with a cardiac illness do not specifically deal with young adults (Fredericks et al., 2012; Foxwell et al., 2013; Herr et al., 2014; Doyle et al., 2015; Li et al., 2015; Tully et al., 2015; Ooi et al., 2016; Le et al., 2018), and studies that do usually focus on patients coping with congenital heart diseases (Van Rijen et al., 2004, 2005a,b; Fredriksen et al., 2009; Asp et al., 2015; Uzark et al., 2015, 2016; Jackson et al., 2017; Abda et al., 2018; Andonian et al., 2018; Grady et al., 2018; Monti et al., 2018).

Therefore, the aim of this systematic review is to identify and gather all the data available on young adult cardiac (YAC) patients (between 18 and 55 years old), coping with all types of cardiac conditions and treatments (coronary, heart failure, arrhythmias, heart transplants, etc.) apart from congenital diseases, regarding their experience and their relationship to their environment, whether social, familial, or medical.

## Methodology

In order to conduct this systematic review, the Preferred Reporting Items for Systematic Reviews and Meta-Analysis guidelines as well as narrative recommendations (Baumeister and Leary, 1997) were followed.

### Search Strategy and Eligibility

We conducted our search on PsycINFO, PubMed, ScienceDirect, and Cochrane library databases, the most relevant ones regarding our topic. Several searches were run on these databases before coming up with the appropriate keywords. The keywords retained related to three categories (both in French and in English): (1) cardiac diseases, (2) young adults, and (3) psychology and patients' experience. The final list was discussed with a cardiologist in order to make sure that no important condition was missing. As we wanted to focus only on cardiac diseases, it was decided not to include the following words (also entered in French): stroke, brain, and cerebral. Our search had to be adapted to the specific constraints of each database. For example, on PsycINFO and PubMed, the search was limited to “human research” (this filter was not available otherwise). On ScienceDirect, the search was limited to titles, abstracts, and keywords among research, review, and data articles. The search terms and equations are available in the Supplementary Material.

Articles published in English or French up to March 1, 2019 were included. We included (1) quantitative, (2) qualitative, and (3) mixed-method articles, which are (4) available online and (5) focusing on the psychological issues (6) of young patients coping with a cardiac illness. Research dealing with patients on a wider age range than 18–55 years old was included only if part of the data concerned our target age range. When necessary, the authors were contacted to request the full texts or to receive more information (mainly regarding the exact age of the participants). Those that were excluded were classified as: (1) non-cardiac-related articles, (2) theses, (3) dissertations, (4) purely medical studies, (5) risk factor studies, (6) congenital heart disease studies, and (7) studies mixing their data with children, (8) adolescents, and (9) adults over 55 years old.

### Data Extraction

**Figure 1:**
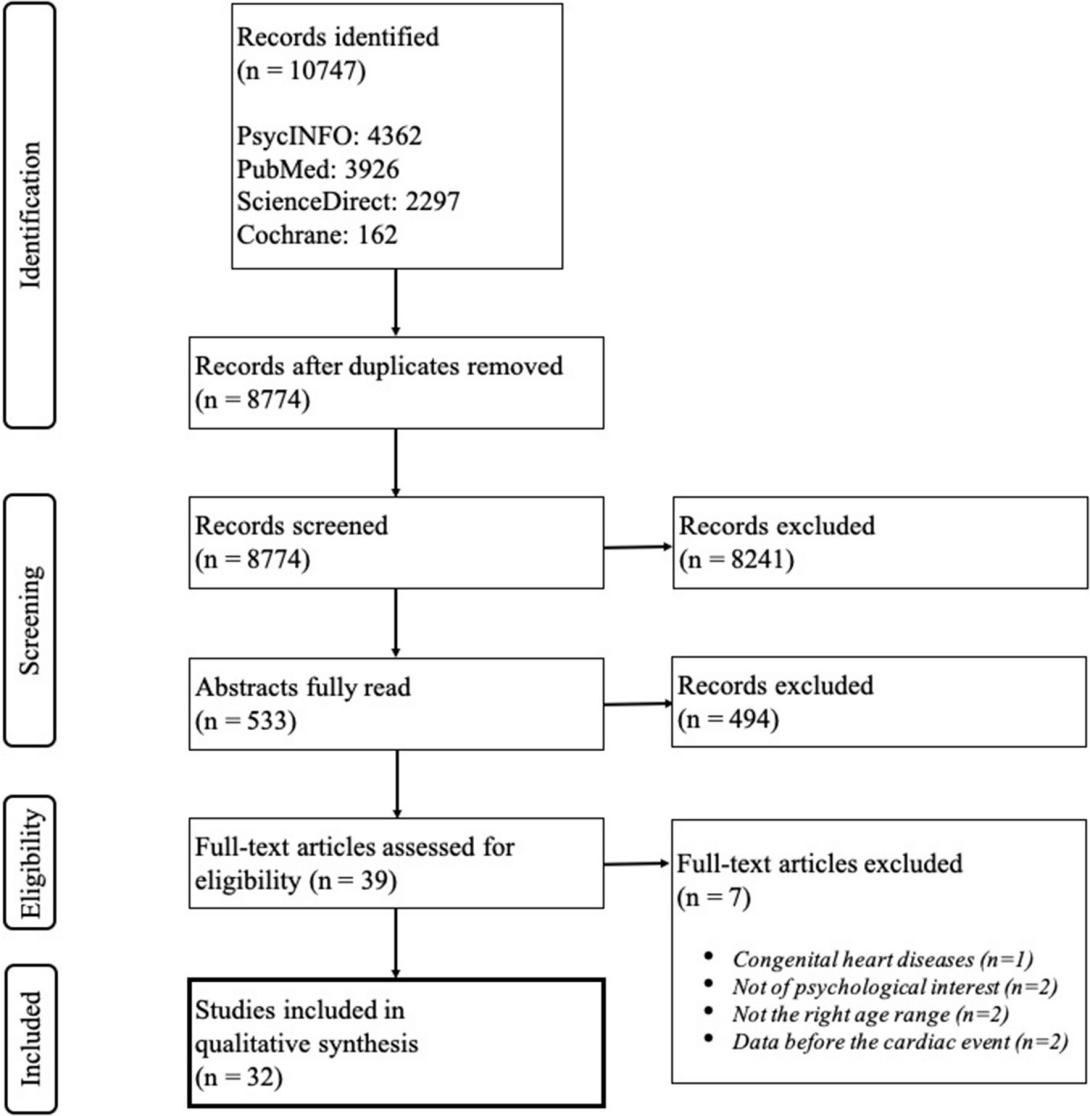
Flow diagram of the study selection.

First, only titles were screened by the main researcher (JJ) to make sure that the data extracted dealt with our inclusion criteria. If there was any doubt, the articles remained in the selection. Then, abstracts were read in order to proceed to a more precise selection. At that stage, a second researcher (CV) screened the articles independently. The eligibility of articles was then discussed by CV and JJ until a consensus was reached. Eventually, full texts were screened to meet the previously stated criteria. JJ took part in each step of the process and Zotero reference manager was used. The flow diagram illustrating the full process described above is presented in Figure 1.

### Quality Assessment

The Crowe Critical Appraisal Tool (CCAT) was used for quality assessment (Crowe and Sheppard, 2011; Crowe et al., 2011, 2012). It is one of the few tools that assess the quality of quantitative, qualitative, and mixed-method studies. The CCAT is composed of eight categories: preliminaries, introduction, design, sampling, data collection, ethical matters, results, and discussion. Each category is rated on five points. The tool provides a grade reaching up to 40, which is then transformed into a quality percentage.

All articles had their quality assessed individually by two authors (JJ + AJ or JJ + CE). They discussed it until a consensus was reached.

## Results

### General Characteristics and Quality Assessment of the Included Studies

**Figure 2:**
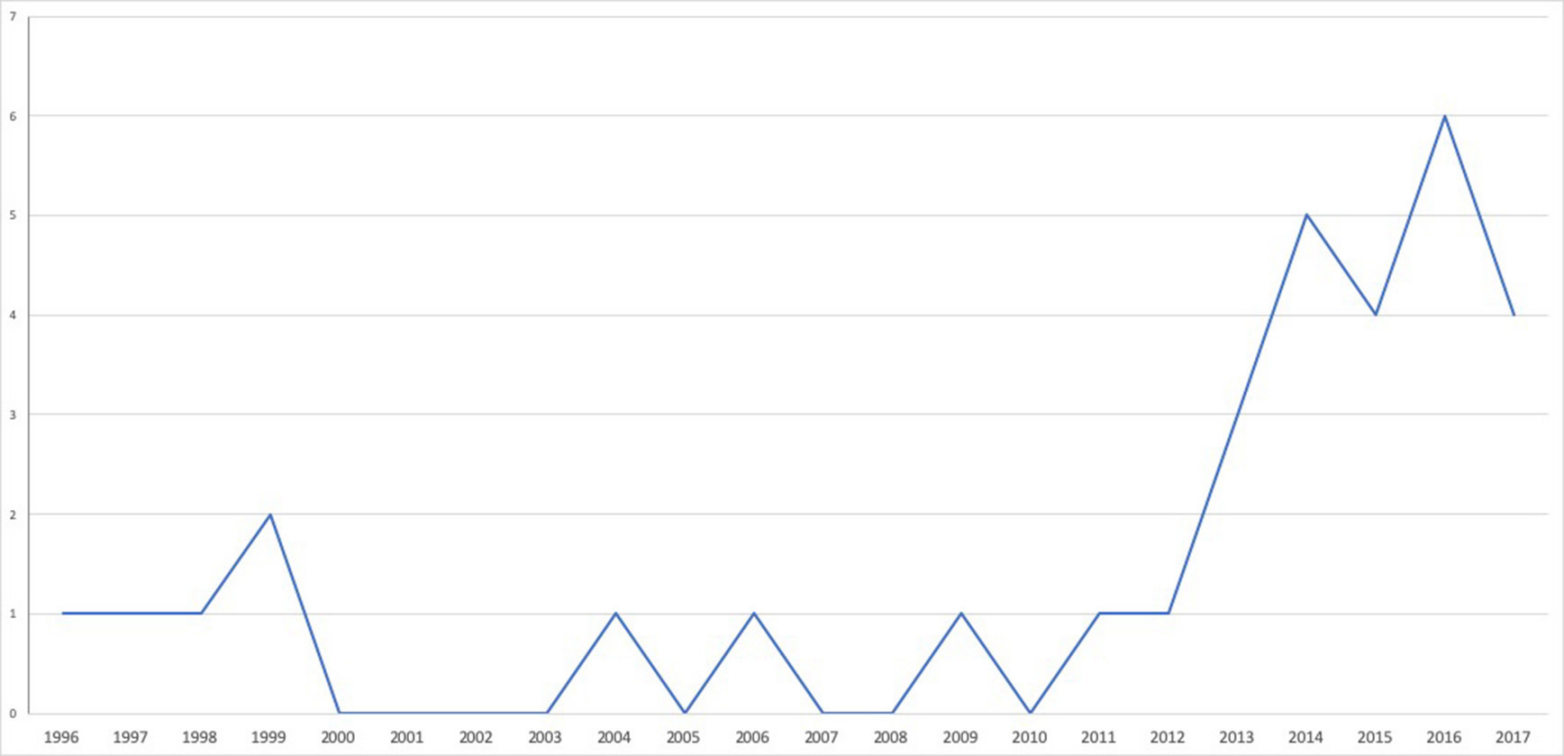
Time distribution of the included published articles on young adult cardiac patients' experience.

Thirty-two studies were included in this review, with a majority of quantitative articles (23) involving between 30 and 3,572 patients. Research was conducted internationally (12 articles) in Europe (nine articles), North America (eight articles), Australia (two articles), and in the Middle East (one article) between 1996 and 2018 (Table 1 and Figure 2). Many articles were published since 2012 because important cohort studies (VIRGO and GENESIS-PRAXY) gave way to numerous publications.

**Table 1:** Characteristics of the included studies.

	***N***
**STUDY DESIGN**	
Quantitative	23
Qualitative	5
Quantitative and qualitative	2
Quantitative and visual^*^	2
**COUNTRY**	
International (multi-country)	12
United States	7
Australia	2
Finland	2
Germany	2
United Kingdom	3
Czech Republic	1
Estonia	1
Israel	1
Sweden	1
**POPULATION**	
Myocardial infarction/acute coronary syndrome	19
Coronary heart/artery disease	7
Cardiac catheterization	2
Cardiac arrest	1
Heart failure	1
Heart transplant	1
Peripartum cardiomyopathy	1

* *Myocardial perfusion imaging*.

All the included participants were patients with a cardiac issue, whether the study focused on their disease (MI and coronary artery disease), their cardiac event (cardiac arrest), or their treatment (catheterization and heart transplant).

The YAC patients represented 66% of the patients from the included studies. The studies mainly focused on individuals after a MI, and most of these patients were coronary patients. Their mean or median age was between 35 and 50 years old. Measures were taken from the time of hospitalization until up to 24 years later.

According to the CCAT's criteria, the overall quality was medium to good (mean 75%), with excellent researchers' inter-rater reliability (0.88). An overview of the articles and their main information, results, and quality data are available in Tables 1, 2.

**Table 2:** Detailed information on the included studies.

**References**	**Range age population at baseline**	***N***	**Mean or median age**	**Pathology/treatment**	**Time of measures**	**Country**	**Study design**	**Quality assessment (researcher 1)**	**Quality assessment (researcher 2)**
Andersson et al. (2013)	23–53	17	45	MI	During the 1st year following their MI	Sweden	Qualitative (interpretative phenomenological analysis, IPA)	85	83
Beckman et al. (2016)	18–55	3,437	47	MI	Within 24 h after MI + 1 month + 12 months	USA and Spain	Quantitative	85	83
Brummett et al. (2004)	<55	No data	No data	CAD (cardiac catheterization)	Hospitalization + 1 month post-catheterization + 3-month intervals over 2 years	USA	Quantitative	63	73
Bucholz et al. (2014)	18–55	3,432	48	MI	Initial hospitalization + 1 month + 12 months	USA and Spain	Quantitative	88	83
Deasy et al. (2012)	29–41	56	35	Cardiac arrest	5 years after the event (range 2.7/8.6 years)	Australia	Quantitative	75	80
Dostálová et al. (2017)	<45	76	No data	MI	<5 years after the event	Czech Republic	Quantitative	60	58
Dreyer et al. (2015)	18–55	3,501	47	MI	Hospitalization, 1 and 12 months post-AMI	USA and Spain	Quantitative	83	88
Dreyer et al. (2016)	18–55	436	No data	CAD (pre-MI)	Hospitalization	USA and Spain	Quantitative	83	85
Hinz et al. (2011)	30–39/40–49	652	No data	MI (or bypass)	At the beginning of their rehabilitation	Germany	Quantitative	70	75
Joubert et al. (2013)	<55	30	47.7	MI	Hospitalization, 3 and 6 months	Australia	Quantitative and qualitative	63	65
Koutrolou-Sotiropoulou et al. (2016)	>18	116	36	Peripartum cardiomyopathy (HF)	0.2 to 24 years after the event	USA	Quantitative and qualitative	65	68
Lacharity (1999)	31–47	11	40	CAD	4 months to 10 years after the event	USA	Qualitative (ethnographic)	70	65
Lavie and Milani (2006)	22–54	104	48	CAD	Baseline 2–6 weeks after CAD event + 1 week later	USA	Quantitative	60	75
Leung Yinko et al. (2014)	18–55	1,123	49	ACS	Baseline (48 h after admission) + 1 month + 6 months + 12 months	Canada, USA, and Switzerland	Quantitative	73	80
Leung Yinko et al. (2015)	18–55	740	50	ACS	48 h after admission + 1 year later	Canada, USA, and Switzerland	Quantitative	73	73
Lindau et al. (2014)	18–55	3,498	48	MI	Hospitalization + 1 month post-MI	USA and Spain	Quantitative	83	83
Lindau et al. (2016)	18–55	2,802	48	MI	Hospitalization + 1 month post-MI + 1 year	USA and Spain	Quantitative	80	83
Lukkarinen and Hentinen (1997)	35–54	No data	No data	CHD	1 or 2 years after medication prescribed for group 1; right before the surgery in the other groups	Finland	Quantitative	65	65
Lukkarinen and Hentinen (1998)	35–54	80	No data	CAD	1 or 2 years after medication prescribed for group 1; right before the surgery in the other groups	Finland	Quantitative	70	70
McAnirn et al. (2015)	35–47	7	42	MI	6–8 weeks after the event	UK	Qualitative (Heideggerian phenomenological)	75	78
Merritt et al. (2017)	29–44	10	40.1	MI	116 mean days since MI	UK	Qualitative (IPA)	90	88
Pelletier et al. (2016a)	42–54	909	48	ACS	Hospitalization (within 24 h of admission) + 12 months	Canada, USA, and Switzerland	Quantitative	70	75
Schweikert et al. (2009)	45–54	No data	No data	MI	7.4 median years since first MI	Germany	Quantitative	80	85
Shah et al. (2014)	<55	851	No data	CAD (angina, MI, and HF)	The day of or before cardiac catheterization	USA	Quantitative	78	83
Uusküla (1996)	30–44	64	37.3, 38.6	MI	Between 4 and 7 days after infarction	Estonia	Quantitative	55	50
Vaccarino et al. (2014)	38–50	49	No data	MI	Within 6 months (5 months median time) after the event	USA	Quantitative and visual	73	78
Vaccarino et al. (2016)	34–50	68	No data	CHD	At least 1 week after the event	USA	Quantitative and visual	80	78
Waldron et al. (2017)	19–29	9	No data	HT (dilated cardiomyopathy, restrictive cardiomyopathy, and previous medical treatments)	7 months to 9.5 years (mean 3.6 years) after the event	UK	Qualitative (IPA)	88	90
Wong et al. (2013)	20–39/40–49	658	No data	HF (ICM, valvular, and ischemic HD)	At least 4 weeks after the event	USA and Canada	Quantitative	68	65
Xu et al. (2015)	18–55	3,572		MI	Baseline + 1 month	USA, Spain, and Australia	Quantitative	75	85
Xu et al. (2017)	18–55	3,509	47	MI	Screening at baseline 3 days after admission + 1 month + 12 months	USA, Spain, and Australia	Quantitative	85	90
Zipori-Beckenstein et al. (1999)	18–55	57	No data	Cardiac catheterization	Right before catheterization and 6 weeks later	Israel	Quantitative	63	63

*MI, myocardial infarction; CHD, coronary heart disease; CAD, coronary artery disease; ACS, acute coronary syndrome; HF, heart failure; ICM, idiopathic cardiomyopathy; HT, heart transplant; HD, heart disease*.

### Themes

Each article was thoroughly analyzed and its data of interest was extracted. Data were gathered in themes and then clustered into superordinate themes.

Five main subjects of interest were revealed: (1) emotional states, (2) quality of life, (3) adjusting to the medical environment, (4) social life, and (5) identity. Each theme included different subthemes that are presented below.

The questionnaires used in each quantitative study are presented in Table 3. The themes explored in each qualitative study are presented in Table 4.

**Table 3:** Questionnaires used in each quantitative study.

**References**	**Depression**	**Anxiety**	**Stress**	**Emotional distress**	**Health-related quality of life**	**Physical functioning**	**Sexuality**	**Interactions with medical professionals**	**Health behavior change**	**Money**	**Social support**	**Work**
Beckman et al. (2016)	PHQ-9		PSS-14		SF-12 and SAQ	SF-12			SD	QC	ESSI	
Brummett et al. (2004)			PSS-10									
Bucholz et al. (2014)	PHQ-9				SF-12 and SAQ	SF-12					ESSI	
Deasy et al. (2012)	EQ-5D	EQ-5D			EQ-5D and SF-12	EQ-5D and SF-12					GOS-E and SF-12	SF-12 and SD
Dostálová et al. (2017)							IIEF-5	SAQ	Biological measures of statins, beta blockers, ACEi/ARBs, and antiplatelet therapy			
Dreyer et al. (2015)	EQ-5D	Item in sex questionnaire			SF-12, SAQ, and EQ-5D	EQ-5D and SF-12		SAQ				SD
Dreyer et al. (2016)	EQ-5D	Item in sex questionnaire			SF-12, SAQ, and EQ-5D	EQ-5D and SF-12		SAQ				SD
Hinz et al. (2011)	HADS	HADS										
Joubert et al. (2013)	CDS				SF-12	SF-12					SF-12 and CPIEAS	
Koutrolou-Sotiropoulou et al. (2016)	QC			QC	QC							
Lavie and Milani (2006)	SF-36	SF-36		KSQ and SF-36	SF-36							
Leung Yinko et al. (2014)	HADS	HADS	Unclear		SF-12 and SAQ	SF-12					ESSI	SD
Leung Yinko et al. (2015)	HADS	HADS							FFQ, GSLPAQ, smoking characteristics, and alcohol consumption habits			
Lindau et al. (2014)	PHQ-9		PSS		SF-12	SF-12	Items adapted from prior large-scale interviewer-administered studies of adult sexuality	QC				
Lindau et al. (2016)	PHQ-9		PSS				Items adapted from prior large-scale interviewer-administered studies of adult sexuality	QC				
Lukkarinen and Hentinen (1997)	NHP			NHP	NHP						NHP	SD
Lukkarinen and Hentinen (1998)	NHP			NHP	NHP						NHP	SD
Pelletier et al. (2016a)	HADS	HADS	QC			Unclear						QC
Schweikert et al. (2009)	EQ-5D	EQ-5D	Unclear		EQ-5D and EQ-VAS	EQ-5D						
Shah et al. (2014)	PHQ-9											
Uusküla (1996)		PARQ		Type A Finnish scale								
Vaccarino et al. (2014)	BDI-II and SCI	STAI-T/S and visual analog ratings of anxiety	SUDS, PSS, blood pressure, and heart rate								ESSI	
Vaccarino et al. (2016)	BDI-II and SCI	STAI-T/S	SUDS, PSS, blood pressure, and heart rate									
Wong et al. (2013)					MLHFQ				Number of pills taken as prescribed			
Xu et al. (2015)	EQ-5D		PSS-14, PSS-10, ISLES	SF-12	SF-12, SAQ, and EQ-5D	EQ-5D and SF-12				QC	ESSI	QC
Xu et al. (2017)	EQ-5D		PSS-14, and ISLES		SAQ						ESSI	QC
Zipori-Beckenstein et al. (1999)		Teichman's questionnaire of trait and state anxiety and QC	QC			QC		QC				

*SCI, Structured Clinical Interview for DSM IV; CDS, Cardiac Depression Scale; NHP, Nottingham Health Profile; SUDS, Subjective Units of Distress Scale; PSS, Cohen's Perceived Stress Scale; ISLES, INTERHEART Stressful Life Events Scale; KSQ, Kellner Symptom Questionnaire; SAQ, Seattle Angina Questionnaire; GOS-E, Glasgow Outcome Scale Extended; SF-12, Medical Outcomes Study Short-Form General Health Survey; HADS, Hospital Anxiety and Depression Scale; EQ-5D, EuroQoL 5 Dimensions; BDI-II, Beck Depression Inventory; IIEF-5, International Index of Erectile Function; FFQ, Food Frequency Questionnaire; GSLPAQ, Godin–Shephard Leisure-Time Physical Activity Questionnaire; CPIEAS, Computerized PIE Assessment Schedule; MLHFQ, Minnesota Living HF Questionnaire; QC, questionnaire created for the study; SD, sociodemographic questionnaire*.

**Table 4:** Themes explored in each qualitative study.

**References**	**Depression**	**Anxiety**	**Stress**	**Emotional distress**	**Physical functioning**	**Sexuality**	**Coping**	**Interactions with medical professionals**	**Health behavior change**	**Money**	**Social support**	**Parenthood**	**Work**	**Identity**
Andersson et al. (2013)		x	x	x	x	x	x		x	x	x	x	x	x
Joubert et al. (2013)			x	x			x	x					x	
Koutrolou-Sotiropoulou et al. (2016)				x				x					x	
Lacharity (1999)	x		x	x		x	x	x	x		x	x	x	x
McAnirn et al. (2015)			x	x			x		x		x	x		x
Merritt et al. (2017)	x	x	x	x					x		x	x	x	x
Waldron et al. (2017)							x		x		x	x	x	x

*x, theme explored in this study*.

#### Emotional States

Regarding emotions and mood, the researchers investigated depression, anxiety, emotional distress, and stress.

##### Depression

Even though depression was investigated in 75% of the included studies (see Table 3), it was usually considered as part of sociodemographic data rather than as a topic of focus in itself. Moreover, almost all studies which included depression rates were quantitative, and many studies used QoL questionnaires to investigate this subject.

Depression rates were inconsistent across studies, ranging from 3 to 90% depending on the sample and the measures used (Lavie and Milani, 2006; Schweikert et al., 2009; Hinz et al., 2011; Deasy et al., 2012; Joubert et al., 2013; Bucholz et al., 2014; Leung Yinko et al., 2014, 2015; Lindau et al., 2014, 2016; Shah et al., 2014; Vaccarino et al., 2014, 2016; Dreyer et al., 2015, 2016; Xu et al., 2015, 2017; Koutrolou-Sotiropoulou et al., 2016; Pelletier et al., 2016a). In the VIRGO studies, which had the most important samples and the highest quality methodology despite the use of different tools, depression rates ranged from 21 to 48%. This wide range was often accounted for by gender, with women being more depressed than men. Several studies compared the depression rates among young patients and among older patients without finding significant differences (Lavie and Milani, 2006; Schweikert et al., 2009; Shah et al., 2014). A year after a cardiac event, the levels of depression decreased (Joubert et al., 2013; Bucholz et al., 2014; Beckman et al., 2016).

Young women were more at risk of being depressed (27%) than older women (12%) (Shah et al., 2014). Women appeared to have higher rates of depression than men after a MI or a coronary disease (Lindau et al., 2014; Vaccarino et al., 2014; Leung Yinko et al., 2015; Xu et al., 2015). This difference between men and women seemed to persist 1 year after the cardiac event (Lindau et al., 2016). However, Pelletier et al. (2016a) made a distinction between feminine *gender* (as a social and cultural condition) and physiologically being a woman. According to them, feminine gender is characterized by specific roles, identity, relationships, and tasks traditionally ascribed to women but which can also be embodied by men. These cultural roles and tasks could predict higher depression rates than biologically being a woman.

In summary, age specificities were observed in women for depression, with younger women being more depressed than older women. Women tended to develop more depressive symptoms than men both during hospitalization and 1 year later.

##### Anxiety

Although anxiety was investigated in 47% of the included studies, it was rarely defined. Some defined it as trait or state.

After a MI, the patients' anxiety was higher than that of the general population both during rehabilitation (Hinz et al., 2011) and 7 years later (Schweikert et al., 2009). Five years after a cardiac arrest, more than half of the individuals included in the study by Deasy et al. (2012) had presented with moderate to severe anxiety. However, apart from the study of Hinz et al. (2011), these samples were too small to allow for generalization.

Depending on the disease and its treatment, the levels of anxiety differed. Right before their cardiac catheterization, state anxiety was lower in the young patients included in the study of Zipori-Beckenstein et al. (1999) than in the older patients (≥55). Within 6 months after a MI, perceived anxiety did not differ between the age groups studied by Vaccarino et al. (2014) (≤50 and >50). However, young coronary patients appeared to score higher on anxiety scales than the other age groups (≥70 and ≥55), whether weeks after a cardiac event (Lavie and Milani, 2006) or years later (Schweikert et al., 2009). Here again the samples were too small to allow for generalization.

After an acute coronary syndrome, the studies showed varying results regarding anxiety levels. According to some authors (Vaccarino et al., 2014), the anxiety levels did not differ between men and women after an acute coronary syndrome, yet another study found that, years later, men had higher levels of anxiety (Schweikert et al., 2009). However, studies on bigger samples suggested that women were more likely to develop anxiety symptoms than men (with 51.7 *vs*. 33.6%, *p* < 0.01) (Leung Yinko et al., 2015). Overall, individuals with feminine-gender characteristics seemed to develop more anxiety than individuals with masculine-gender characteristics, whether they were men or women (Pelletier et al., 2016a).

Therefore, according to the information available, cardiac diseases or their treatment may trigger anxiety that tends to remain over time, but evidence regarding age and sex was inconsistent.

##### Emotional distress

A focus on emotional distress was found in 34% of the included studies. Anger and irritability were often evoked by patients and seemed to be part of their emotional state from their hospitalization until at least a year later, both in quantitative and qualitative studies (Uusküla, 1996; Lacharity, 1999; Lavie and Milani, 2006; Andersson et al., 2013; McAnirn et al., 2015). According to Lavie and Milani (2006), hostility was higher among younger patients (<55) compared to older patients (≥70).

In qualitative studies, a “heightened sense of mortality” was described by the participants throughout the first year after diagnosis (Lacharity, 1999; Andersson et al., 2013; Joubert et al., 2013), although it was described less often by men for whom it was difficult to accept death as a nearby possibility (Merritt et al., 2017). Emotional distress and the heightened sense of mortality were found in several expressions of fear: of dying in their sleep, of dealing with another MI (Andersson et al., 2013), of pain (Joubert et al., 2013), or of other injuries provoked by the disease (Lacharity, 1999; Koutrolou-Sotiropoulou et al., 2016). Worry was part of the young patients' emotional states 1 week after hospitalization in a quantitative study (Uusküla, 1996), and up to 6 months later in a qualitative study (Merritt et al., 2017). The patients also felt guilt and responsibility with regards to their family and to past bad lifestyle choices (McAnirn et al., 2015). These feelings seemed to last up to a year later, according to some high-quality studies (Andersson et al., 2013; Merritt et al., 2017).

Few of these studies compared men and women. Among the quantitative studies, Uusküla (1996) found that women were more subject to irritation, inability to relax, and impatience. Lukkarinen and Hentinen (1997, 1998) found that women had more frequent emotional reactions than men, and in the study by Xu et al. (2015), women tended to develop more severe mental health issues than men during hospitalization. However, several qualitative articles included men only (Merritt et al., 2017) or women only (Lacharity, 1999; Andersson et al., 2013; Koutrolou-Sotiropoulou et al., 2016). Koutrolou-Sotiropoulou et al. (2016) found that few women felt that they had recovered emotionally during the 6 months following their first symptoms and more than half of them never returned to their normal previous emotional level. According to the quantitative study of good quality of Xu et al. (2015), mental functioning was worse among young adult MI patients a year after their hospitalization compared to during their hospitalization.

To conclude, YAC patients expressed emotional distress, with feelings such as hostility and a sharpened fear of death. Women may have a stronger risk of not recovering emotionally from their disease.

##### Stress

Stress was rarely properly defined, even though it was accounted for in 53% of our selection. Most of the articles referred to stress as a risk factor and in relation to work, children, finances, and lifestyle. Informative data were mainly found in quantitative articles. Out of the 17 articles with a focus on stress, “perceived stress” was only investigated in six articles. Stressful life events were separately assessed in two studies. Two studies assessed stress in experimental conditions (Vaccarino et al., 2014, 2016): they used blood pressure and heart rate measures as they intentionally induced mental stress with a standardized public speaking task. Surprisingly, post-traumatic stress was never explored in these studies.

After significant stress during hospitalization for a cardiac event, stress decreased over time, both 1 month later and 2 years later (Brummett et al., 2004; Beckman et al., 2016; Xu et al., 2017). Ischemia due to stress was three times higher among young women than among men (Vaccarino et al., 2014, 2016). Furthermore, perceived stress scores were higher among coronary women compared to the scores of coronary men both during their hospitalization and over a 2-years period (Brummett et al., 2004; Lindau et al., 2014, 2016; Xu et al., 2015, 2017). However, these data are to be taken lightly as the number of YAC patients in these studies was either small or undisclosed.

When young patients were compared to older patients regarding stress, no difference was found during hospitalization or 6 months later (Vaccarino et al., 2014), but the youngest tended to score higher over a 2-years period (Brummett et al., 2004).

From these studies, it appeared that the levels of stress decreased over time, that YAC patients may develop more stress than older patients years after a cardiac event, and that women were often found to be more stressed than men.

#### Quality of Life

Quality of life was explored in 62% of our included articles. These studies examined either health-related QoL or more specific aspects, such as physical functioning or sexuality.

##### Health-related quality of life

Half of the articles corresponding to our inclusion criteria investigated QoL, and they were all quantitative. Out of these 16 articles, quality of life was only properly defined three times: it was defined as the way patients felt, perceived, and defined their health in relation to physical, psychological, and social aspects (Lukkarinen and Hentinen, 1997; Deasy et al., 2012; Leung Yinko et al., 2014). Most of the time, QoL was explained through the tools used and was used as a way to measure the effectiveness of medical interventions.

From the moment of their hospitalization to 7 years later, young adult MI patients presented lower QoL scores than the general population (Schweikert et al., 2009; Leung Yinko et al., 2014). Some patients improved after a year (Bucholz et al., 2014; Beckman et al., 2016), and a small sample improved 5 years after their cardiac arrest (Deasy et al., 2012). Several authors found that being a woman was a risk factor for worse QoL from hospitalization to months and years after (Lukkarinen and Hentinen, 1997; Schweikert et al., 2009; Dreyer et al., 2015, 2016), but according to Leung Yinko et al. (2014), this difference could be attributed to a more feminine gender role, low social support, and high housework responsibilities.

From 1 month to a year after hospitalization, two studies of poor quality found that younger adults had lower QoL scores than older patients (Wong et al., 2013), even though their medical state was not objectively considered as worse (Lukkarinen and Hentinen, 1997).

Moreover, high QoL scores have been shown to be linked to lower depression, anxiety, and stress scores, both when patients were hospitalized and a month later (Leung Yinko et al., 2014; Xu et al., 2015).

Therefore, being a woman and being young may represent a higher risk for low QoL among individuals with a cardiac illness. High levels of QoL seemed to be associated with better mental health. Over time, QoL appeared to improve.

##### Physical functioning

Physical functioning was part of the QoL tools used by the researchers and thus represented an important aspect of QoL and the experience of individuals with a cardiac illness. It was explored in 40% of the studies.

Being young was associated with better health and better chances of recovering from a cardiac intervention, such as cardiac catheterization (Zipori-Beckenstein et al., 1999).

Years after a cardiac arrest, the vast majority of the patients of the study of Deasy et al. (2012) had good mobility, personal care, usual activities, and no pain or discomfort, yet 11% had a severe disability. After a MI, it was found that physical functioning in daily activities improved during the following 3–6 months, offering renewed independence in work-related and daily occupations (Joubert et al., 2013). From a qualitative point of view, during the first year after a MI, the patients described recurrent moments of “overwhelming fatigue,” preventing them from doing anything (Andersson et al., 2013).

Cardiac events and interventions were shown to have physical impacts on young patients, but they tended to disappear. However, studies were too scarce, and too few participants were included to conclude on this aspect.

##### Sexuality

A focus on sexuality was found in 15% of the articles reviewed. The studies of Lindau et al. (2014, 2016) made a substantial contribution to this theme with their important samples and good-quality quantitative methodology.

There was a higher rate of declared sexual problems among patients after a MI than in healthy people of the same age (Lindau et al., 2016). A month after a MI, sexual activity was found to have declined compared to before the infarction but almost completely resumed during the following year (Lindau et al., 2014, 2016). However, one in 10 women and one in 20 men never resumed a sexual activity. The following factors associated with being a “late resumer” were identified: being a woman and in a relationship, of American nationality (as opposed to Spanish), coping with stress, having lost general physical functioning, not communicating with a physician about these issues, and being in the older range of a cohort of individuals 18–55 years old. In this cohort, the incidence of sexual problems was much higher than the incidence of depression (Lindau et al., 2016).

Some discrepancies between countries, regarding the way these issues were addressed by professionals, emerged. Women and Americans were less likely to bring up the subject with their physician, and the discussion was initiated more often by the professional in Spain than in the USA (Lindau et al., 2016). Men did not often dare talk about their erectile dysfunctions, and there was a lack of screening and treatment for this issue (Lindau et al., 2014; Dostálová et al., 2017). The physicians' recommendations were inconsistent: they tended to suggest limiting sexual activity even though it was not related to the patients' state of health (Lindau et al., 2016). Qualitatively, the lack of counseling and advice was a source of dissatisfaction for the patients (Lacharity, 1999).

To sum up, young individuals coping with a cardiac illness encountered diverse sexual problems that were not necessarily addressed by their doctor.

#### Adjusting to the Medical Environment

In addition to focusing on the impact that cardiac diseases may have on YAC patients, some articles were also interested in the way the patients adjusted to the medical environment. They presented the patients' coping strategies, the way the patients changed their health behaviors, the financial barriers that they had to face and their relations with medical professionals.

##### Coping with the disease

Coping strategies were addressed by the patients interviewed in more than half of the qualitative studies. They were only explored in 15% of the overall included studies. The main strategies evoked were:

- *Living in the present*: Even though some patients were struggling, this strategy may be a way of remaining positive about the future (Andersson et al., 2013) and of limiting the impact of the disease on their identities (Waldron et al., 2017).
- *Avoidance*: Some patients tried to avoid thoughts about their own mortality (Waldron et al., 2017), avoided expressing their emotions for fear of the impact on their personal and working relationships, and avoided expressing their sexual needs to their partner (Andersson et al., 2013).
- *Change*: Some patients changed their behaviors to focus on what matters in life and in order to modify the risk factors (Lacharity, 1999). The participants tended to believe that those changes were important and under their control (McAnirn et al., 2015). On the other hand, the patients sometimes felt the urge to go back to their previous lifestyle regarding work, leisure, and relationships to effectively manage their emotions and difficulties (Joubert et al., 2013).
- *Acceptance* of the disease and its inherent physical limitations (Lacharity, 1999).
- *Social support*: Even though the participants looked for social support (emotional and material) and wanted to stay close to their relatives at all times, they sometimes found it difficult to accept help from their friends and family (Lacharity, 1999; Andersson et al., 2013).
- *To take refuge in spirituality* was also seen as a way to gain strength for some patients (Lacharity, 1999).

##### Health behavior change

We define health behavior change as therapeutic adherence and following recommendations regarding medication, diet, physical activity, smoking, alcohol, and drug consumption. This issue was explored in 28% of the included studies, which were of rather good quality and mostly comprised of large samples.

Generally, right after a MI, young patients had a worse health behavior profile than the general population (Leung Yinko et al., 2015). However, for the first 4 months, they were motivated to change (McAnirn et al., 2015) and felt good about implementing a healthier lifestyle (Merritt et al., 2017). These qualitative data matched the quantitative information: some patients thus improved their lifestyle regarding physical activity, alcohol consumption, and dietary changes and were more compliant to medication (Leung Yinko et al., 2015; Dostálová et al., 2017). Others maintained the healthiest possible lifestyle but with “planned exceptions” in order to cope with their frustration (Lacharity, 1999). On the other hand, adopting a healthy behavior regarding diet and physical activity was qualified as a constant struggle that could be improved with professional help (Andersson et al., 2013). Medication was also associated with negative feelings, such as the loss of independence (Merritt et al., 2017). To that extent, in quantitative studies, the youngest heart failure patients were less likely to adhere to medication and dietary changes (Wong et al., 2013). It also appeared that women had more barriers to medication, smoking cessation, and dietary changes than men (Leung Yinko et al., 2015; Beckman et al., 2016).

After a cardiac event, contrasted health behavior profiles arose: they were negatively invested by some individuals who see medical recommendations as constraints, whereas some transformed these obligations into a renewed, healthier, and rewarding lifestyle.

##### Financial barriers

Financial barriers were only mentioned in 9% of the included articles. These studies were all of good quality and had good methodology, with large samples.

After a MI, one third of the young patients had trouble accessing healthcare services (follow-up visits and rehabilitation) because of financial barriers and 20% were not able to get access to medication. These rates were higher than in older MI patients. These financial barriers led to lower QoL, more depressive symptoms, and higher perceived stress (Beckman et al., 2016).

Even though not having enough money was linked to more stress for men (Xu et al., 2015), women were more at risk of having financial problems. They considered their medical costs a moderate or severe burden, especially when living with children at home (Xu et al., 2015; Beckman et al., 2016). In a qualitative study, some young patients described having to face such important financial problems that they borrowed money from their relatives or sold their personal possessions (Andersson et al., 2013).

All in all, the specific situation of younger patients (with children and without jobs) led to financial difficulties which could prevent them from being compliant to their treatment. However, we must bear in mind that this issue is strongly dependent on the healthcare system unique to each country and therefore cannot be generalized.

##### Interactions with medical professionals

Regarding interactions with the medical world, communication between patients and physicians and the delivery of sufficient and adequate medical information were the two main topics that emerged. This theme appeared in 31% of articles. Only 26% of the women interviewed by Koutrolou-Sotiropoulou et al. (2016) were satisfied with the counseling that they received from their physician. One third reported not receiving adequate counseling. Not receiving information on their disease, its causes, consequences, and prevention of recurrence brought large amounts of fear (Andersson et al., 2013; Joubert et al., 2013). Quantitatively, Zipori-Beckenstein et al. (1999) also found that low levels of anxiety were correlated with high positive expectations that produced better treatment satisfaction.

The patients sometimes found it difficult to gain access to medical support (Joubert et al., 2013) and when they did, for instance in rehabilitation, they sometimes felt a lack of attention to their actual needs (Andersson et al., 2013). Dissatisfaction was also expressed when healthcare professionals were too demanding, resulting in a feeling of stress.

In quantitative studies, women showed less treatment satisfaction than men (Dreyer et al., 2015, 2016). They felt less counseled than men 1 month after their hospitalization, even though they would have considered it appropriate for their physician to do so (Lindau et al., 2014). A year after hospitalization, both men and women had received more sexual counseling than a month after hospitalization, even if it remained scarce (Lindau et al., 2016). For men, communication about erectile dysfunctions did not happen often and was deemed insufficient (Dostálová et al., 2017).

Even though the quality and the samples of the studies varied, they agreed on the fact that many patients were dissatisfied with the communication received from healthcare professionals, specifically regarding information given by physicians.

#### Social Life

Some studies took into account the patients' personal environment and addressed the social aspects of young individuals' lives when coping with a cardiac illness, such as perceived social support and work.

##### Social support

The studies investigated different social aspects: activities, support, isolation, dependence, and the social impact of the disease. These were investigated through dedicated questionnaires or through QoL subscales (see Table 3) and were a topic for almost half (47%) of the included studies.

Lack of social activities (such as visiting friends or relatives) was associated with difficulties, such as emotional and physical health problems (Joubert et al., 2013). Lack of social support was correlated to lower QoL, lower mental functioning, and more depressive symptomatology (Bucholz et al., 2014; Leung Yinko et al., 2014). Even though these problems generally did not last a year after the cardiac event, in one study with a small sample (Joubert et al., 2013), QoL was impacted throughout the year following a MI. In another better-quality study with a bigger sample, this result was more significant among women because of low social support (Leung Yinko et al., 2014).

Patients coped with limitations in their social activities (Joubert et al., 2013) and had to carefully plan their outings to save energy (Andersson et al., 2013). They also coped with isolation, which was more prevalent among younger patients (Lukkarinen and Hentinen, 1997), but these data have to be confirmed.

In qualitative studies, friends and family were seen as one of the most important sources of support, and their support was preferred to rehabilitation (Lacharity, 1999; Joubert et al., 2013; McAnirn et al., 2015). However, it was difficult for many patients to accept the change in their close relationships arising from their increased dependence (Lacharity, 1999; Andersson et al., 2013; Joubert et al., 2013). They sometimes felt their relatives were overprotecting them and prevented them from participating in physical activities that patients knew (from professionals) they were allowed to perform. This resulted in anxiety, avoidance, and conflicts (Merritt et al., 2017; Waldron et al., 2017). The patients sometimes also felt misunderstood by people with whom they used to partake in unhealthy behaviors (such as smoking and drinking) and expressed the need to meet other young patients (Merritt et al., 2017).

Low social support was therefore associated with psychosocial difficulties (regarding health, mental functioning, depression, anxiety, and conflicts). Over time, only the association between low social support and QoL remained. Young patients coped with isolation and found it difficult to accept the switch toward dependent relationships despite social support being very important to them.

##### Work

Work status or thoughts about work were assessed in almost half (47%) of the articles through sociodemographic data, questionnaires, and qualitative interviews. However, even though the nature of work was indicated, it was not often explored in detail after the disease onset. More often, it was investigated as a risk factor before the disease onset.

While heart-transplanted patients all went back to work years after their surgery (Waldron et al., 2017), coronary patients did not (Lacharity, 1999). Only 68% of adults having coped with a cardiac arrest were working 5 years later (Deasy et al., 2012) and 28% of the women coping with peripartum cardiomyopathy discontinued their job (Koutrolou-Sotiropoulou et al., 2016). The main motivations to go back to work were financial and a will to return to “normality” (Lacharity, 1999).

Qualitative studies showed that work became worrisome for patients after a MI: they expressed fear of not being efficient, of losing or not getting their job back, of financial difficulties, and of stress (Andersson et al., 2013; Joubert et al., 2013). Some saw this crisis as an opportunity to rethink their priorities by finding employment more suitable to their new situation, mainly looking for more meaning and less stress (Merritt et al., 2017).

The YAC patients showed no pattern regarding going back to work. The issue of work was a source of stress and anxiety for many. For some, the disease acted like a wake-up call, leading them to orient toward more meaningful work. However, the lack of large samples and quantitative data pleads for more research in this area.

#### Identity

After a cardiac event, one's identity is challenged: “you're just not the same” (Lacharity, 1999). Individuals become patients, with new challenges to face and a new equilibrium to find (Waldron et al., 2017). Through this experience, some of them reflected on their status as parents (Lacharity, 1999; Andersson et al., 2013; Merritt et al., 2017; Waldron et al., 2017).

These topics were a subject of interest in 16% of the included studies and were only explored in qualitative articles.

##### Parenthood

On the one hand, the disease or its treatment was sometimes the starting point for the desire to have children. It was described as a new motivation to start a family and was occasionally discussed with professional caregivers (Merritt et al., 2017; Waldron et al., 2017). On the other hand, other patients feared parenthood as they became aware of their disease and worried about leaving their children behind (Andersson et al., 2013; Merritt et al., 2017). They also worried about the impact of the illness on their children: they feared not being able to meet their offspring's expectations, not being able to fulfill their duty as parents, and seeing them cope with the same disease (Lacharity, 1999; Andersson et al., 2013).

##### New challenges

Faced with a cardiac disease, the participants started feeling different from their former selves (Lacharity, 1999): a body described as unfamiliar and weak resulted in decreased self-confidence in front of such a life-threatening situation (Andersson et al., 2013; McAnirn et al., 2015).

Among coronary and heart-transplanted patients, some referred to this search for new equilibrium as a “fight” (Andersson et al., 2013; Waldron et al., 2017). Men felt less masculine, strong, and independent (Merritt et al., 2017; Waldron et al., 2017) and women found it hard to see their physical attractiveness lessen, partly due to the weight gain caused by their treatments (Waldron et al., 2017).

The heart-transplanted patients described a “paradoxical position”: they wanted to move away from the disease while still having to undergo medical routines (Waldron et al., 2017). However, an old study, which was mediocre in quality, reported that coronary women tended to accept their illness and understand their limits (Lacharity, 1999). Additionally, both coronary and heart-transplanted patients felt different from older cardiac individuals: they felt like they should not be here precisely because they were too young to experience this (Waldron et al., 2017) or because they may have had different needs (Andersson et al., 2013).

##### New meanings

New meanings were evoked in two studies of good quality (Andersson et al., 2013; Waldron et al., 2017). In these studies, individuals coping with a cardiac illness viewed their new situation as a “second chance” or a “gift of life.” For transplanted individuals specifically, there was a feeling of debt to donor families and they had high expectations to become even “better than normal.” According to the authors, the patients had difficulty grasping the idea that this feeling of normality was more a process than an instant state resulting from the surgery.

For some patients, these experiences resulted in the search for a new meaning of life, with material possessions, work, and risk taking becoming less valued than quality time with family members. Aspirations in these areas were challenged. Although this was scary for some individuals even years after, others found some sort of stability after a year.

The identities of YAC patients were characterized by a struggle with their previous concept of normality, a new look upon their body, and the sense of being different from older patients. Moreover, parenthood was not taken lightly. Those who already had children started worrying about the impact of the disease on their offspring, while many of those who were not yet parents built a parental project in spite of their fears.

## Discussion

This systematic review showed that YAC patients are a specific group with specific needs. Compared to older patients coping with a cardiac illness, YAC patients sometimes had higher levels of depression, anxiety, and stress. They had lower QoL and coped with social isolation and financial barriers. Other issues, such as work and parenthood, were specific to this age range. Furthermore, these individuals felt different from older patients with a cardiac illness: they felt too young to experience these issues and had different needs compared to the older patients. Women represented a special sub-group more at risk of developing depression symptoms, stress, emotional distress, and lower QoL.

The results from this systematic review established that the levels of depression are sometimes high among YAC patients (Joubert et al., 2013) but, similarly to previous studies with wider age ranges, age comparisons led to unconvincing results (Lavie and Milani, 2006; Schweikert et al., 2009; Shah et al., 2014).

Our results highlighted the link between QoL, depression, anxiety, and stress (Leung Yinko et al., 2014; Xu et al., 2015). In the literature, depression was found to be the strongest predictor of young adult coronary patients' QoL (Lane et al., 2001, as cited by Vaccarino et al., 2019). The QoL of YAC patients seemed low and lower than that of the healthy population (Schweikert et al., 2009; Wong et al., 2013; Leung Yinko et al., 2014). A recent systematic review and meta-analysis showed that this was also true for older adult patients (mean age = 61 years old) with coronary heart disease (Le et al., 2018). This may not be the case for younger congenital heart disease patients (7–17 years old) who had QoL levels identical to those of healthy controls (Reiner et al., 2019). However, in the context of implantable cardioverter defibrillators (ICD), younger patients were found to cope with a lower QoL than older patients (Friedmann et al., 2006). This could be explained by a feeling of lost independence in the young, whereas the older patients saw this treatment as a way to extend their life (Arteaga and Windle, 1995, as cited by McDonough, 2009). It could also be argued that, in cardiac settings, the young are struck in a moment of their life perceived as disease-free, while older patients may accept the disease and its treatments as the growing burden of age and impairment. This could explain the differences between young and old patients with a cardiac illness regarding QoL.

Our findings demonstrated the importance of social support for YAC patients, especially from close relatives (Lacharity, 1999; Joubert et al., 2013; Bucholz et al., 2014; Leung Yinko et al., 2014; McAnirn et al., 2015). For YAC patients, a lack of social support was associated with health, emotional, mental, and depressive issues and lower QoL (Joubert et al., 2013; Bucholz et al., 2014; Leung Yinko et al., 2014). These results are consistent with the literature in older patients for whom the presence of friends, family, and partners seems crucial after the appearance of a cardiac disease (Kristofferzon et al., 2008; Bertoni et al., 2015; Ooi et al., 2016). According to the findings, young patients (≤40 years old) may be more at risk of isolation after the implant of an ICD (Dubin et al., 1996; Sears et al., 2009; Larimer et al., 2016). This leads us to underline the importance of developing research among patients and their relatives, especially couples, to improve their emotional and physical outcomes (Sher et al., 2014; Varela Montero and Barrón López de Roda, 2016; Lyons et al., 2017; Trump and Mendenhall, 2017).

Our data underlined issues already expressed in previous studies. It was found that even though YAC patients needed support from their friends and family, it was sometimes difficult for them to accept their assistance (Lacharity, 1999; Andersson et al., 2013; Joubert et al., 2013). Similarly, while many patients (47–90 years old) received support from their network, they found it difficult to communicate with them after a MI (Kristofferzon et al., 2007). We also found that many patients were unhappy about the support received from professionals (Andersson et al., 2013; Joubert et al., 2013; Koutrolou-Sotiropoulou et al., 2016; Ooi et al., 2016), especially regarding sexual issues (Lacharity, 1999; Lindau et al., 2014, 2016; Dostálová et al., 2017). Indeed the patients expressed the need for sexual counseling after cardiac issues and wanted professional health caregivers to provide for it (Byrne et al., 2013; Mc Sharry et al., 2016; Ooi et al., 2016). This finding is not specific to cardiac settings: it is common that professionals do not feel eligible to address these issues and instead feel uncomfortable (Mellor et al., 2013).

Regarding these topics, we can conclude on two aspects: Firstly, training healthcare providers in efficient communication with patients could improve patient satisfaction. To that extent, more information regarding the key issues expressed by the patients is needed. Secondly, in order to increase social support, it could be argued that systemic patient and relative care would be beneficial as well (Liljeroos et al., 2017; Smith and Baucom, 2017).

Studies highlighted the way in which YAC patients' identities were challenged because of their new perception of their bodies (Lacharity, 1999; Andersson et al., 2013; McAnirn et al., 2015; Merritt et al., 2017), their desire to return to normality while having to change habits (Waldron et al., 2017), and their feeling of difference from older patients with a cardiac illness (Andersson et al., 2013; Waldron et al., 2017). The new perception of themselves also challenged their equilibrium and status, such as parenthood (Lacharity, 1999; Andersson et al., 2013; Merritt et al., 2017; Waldron et al., 2017). While our findings highlighted the way the participants attribute new meaning to life after a cardiac issue, other studies went further by reporting a form of resilience or personal growth after a MI (Kristofferzon et al., 2008), among teenagers with a congenital disease (Apers et al., 2016) or people living with an ICD (Ooi et al., 2016). As in our review, most other results focused on the physical difficulties and especially perceptions of the injured body. These provoked impairment and feelings of dependence and uselessness, with social limitations as a result (Vazquez et al., 2008; Allison and Campbell, 2009; McDonough, 2009; Ooi et al., 2016; Jackson et al., 2017; Chong et al., 2018). Weakness was especially incompatible with the vision men had of themselves (Allison and Campbell, 2009). Additionally, young patients with a cardiac illness felt different from older populations, having to deal with specific issues related to their age, such as childbearing and financial concerns (Allison and Campbell, 2009; McDonough, 2009; Ooi et al., 2016).

These concerns might explain, to some extent, the non-adherence found among young patients with a cardiac illness in our included studies. Indeed young patients tend to have a less stable professional life, with college to attend or children to raise. Moreover, the literature highlights associations between non-adherence and younger age (Tumin et al., 2017). To our knowledge, while the rate of non-adherence among YAC patients has not been studied, meta-analyses show that 20 to 30% of older coronary patients do not take their medication (Crawshaw et al., 2016, 2017). Whatever their age, the common difficulty lies in time: even when coronary patients are compliant right after the event, as years go by their adherence seems to decrease (Baldacchino, 2011; Andersson et al., 2013; Leung Yinko et al., 2015). Studies also highlight that non-adherence is linked to depression (Crawshaw et al., 2016, 2017; Vaccarino et al., 2019). Therefore, interventions need to target health behavior changes right after a cardiac event, with regular reminders to increase or maintain adherence.

Finally, women emerged as a specific sub-group in this systematic review. Women seem more at risk of developing depression (Lindau et al., 2014, 2016; Vaccarino et al., 2014; Leung Yinko et al., 2015; Xu et al., 2015), poor QoL (Lukkarinen and Hentinen, 1997; Schweikert et al., 2009; Dreyer et al., 2015, 2016), and poor psychological and physical outcomes (Uusküla, 1996; Lukkarinen and Hentinen, 1997, 1998; Brummett et al., 2004; Lindau et al., 2014; Vaccarino et al., 2014; Leung Yinko et al., 2015; Xu et al., 2015, 2017; Pelletier et al., 2016a). This is consistent with the literature in other age ranges (Mallik et al., 2006; Vazquez et al., 2008; Sears et al., 2009; Ingles et al., 2013; Doyle et al., 2015; Pelletier et al., 2016b; Sabbag et al., 2017; Benderly et al., 2019; Vaccarino et al., 2019). Some studies suggest that mental health issues could partly be explained by the impact on body image that seems to be more difficult for women (Vazquez et al., 2008; Starrenburg et al., 2014; Ooi et al., 2016) and by the multiple social roles women have to handle, such as parenting and housekeeping, as well as developing their career (Vazquez et al., 2008).

Although this systematic review highlighted several specificities of YAC patients, it also brought to light the difficulties encountered to aggregate data on this subject. Indeed different methods (quantitative, qualitative, mixed) and also different tools (specific validated questionnaires, more general ones with only some items of interest, or questionnaires created for the study) were used to assess the psychosocial variables. Furthermore, different populations (MI, coronary artery disease, cardiac arrest, catheterization, and heart transplant) and different times of measures (during hospitalization or years later) were studied. Therefore, it was difficult to compare the data. It appears that research on patients with a cardiac illness is dominated by quantitative studies, with significant focus on MI (see Table 2). This was partly due to a major group of research, the VIRGO studies, that accounted for a quarter of these articles (Bucholz et al., 2014; Lindau et al., 2014, 2016; Dreyer et al., 2015, 2016; Xu et al., 2015, 2017; Beckman et al., 2016). Furthermore, studies on YAC patients seem to be more about “young adults” and middle-aged adults, who are probably easier to include because they are more numerous in cardiac settings than “emerging adults” (Arnett, 2000).

To our knowledge, this is the first systematic review summarizing the psychological experiences of YAC patients. It shows their specific needs and identity compared to older cardiac individuals. However, there are several limitations to this review. Firstly, a majority of the studies reviewed concerned patients with coronary diseases. Our results might then be a more accurate description of coronary patients rather than of all individuals with a cardiac illness. However, our aim was to develop an overview of multiple cardiac diseases, and while the distinction between heart diseases is essential for professionals, it may not be for patients. Moreover, apart from heart-transplanted patients, few differences were found between patients with different cardiac issues. Therefore, regrouping patients according to their age, not their disease, may be more relevant.

Secondly, we were not able to include specific information regarding patients living with ICD or valvular prostheses as the relevant studies identified always focused on participants with congenital heart diseases. However, almost all the references of our included articles were checked for this subject matter. Therefore, it is highly probable that all the relevant research studies were included.

Lastly, the data selection was conducted by the main researcher only. However, the risk of selection bias was reduced thanks to two independent authors performing the study selection, and frequent discussions took place on the data, themes, and interpretation within the author's team. Moreover, the quality of each study was assessed by two researchers. Eventually, the results seemed to represent the healthcare system of each country where recruitment took place well since we found, for instance, financial barriers in the USA where patients spend a lot for their medical expenses.

## Conclusion

YAC patients seem to constitute a specific population among individuals with a cardiac illness. This systematic review presented the psychosocial experience expressed by these patients who cope with symptoms of depression, stress, anxiety, low QoL, and social isolation. Several of these outcomes may be more difficult to endure for YAC patients than for other age groups due to their life context with careers to develop, children to raise, and financial responsibilities while coping with the disease. In spite of the specific threats inherent to each cardiac disease, YAC patients are to be considered with their own identity and challenges. Moreover, young women are a particularly fragile group among patients with a cardiac illness. Therefore, psychosocial interventions targeting YAC patients to improve their mental health and adherence should be trialed. Practice and research implications are available in Box 1. Interventions could focus on the exploration of emotions, beliefs, and the feeling of self-efficacy (Riegel et al., 2006; Riegel and Dickson, 2016). It would also be interesting to test whether or not interventions specific to their age group would be beneficial to YAC patients in terms of mental health and quality-of-life improvements, with a special focus on women. Future studies should also include a focus on post-traumatic stress disorder as, despite the vast literature pointing out its prevalence among cardiac patients (Vilchinsky et al., 2017), it was not addressed in the included studies. Research could also include caregivers as they were found to be impacted by the disease (Trump and Mendenhall, 2017); thus, they may influence both the well-being and the adherence of patients (Sher et al., 2014; Varela Montero and Barrón López de Roda, 2016; Lyons et al., 2017).

BOX 1 **Practice and research implications**

**Implications for practice**

- Consider young patients coping with a cardiac illness as a specific population, different from teenagers and older adults.
- Explore adherence and present it as a way to a healthier and a more meaningful way of life.
- Help young adult cardiac (YAC) patients get in touch with other young patients and promote communication with their families and professional caregivers.
- Accompany YAC patients in their new identity and help them find effective ways of coping.
- Provide sexual counseling.

**Implications for research**

- Consider YAC patients as a specific population.
- Study other cardiac diseases than coronary/myocardial infarction.
- Pay attention to the specificities of women.
- Identify the most efficient coping strategies to enhance mental health issues and quality of life.
- Test interventions (psychoeducation and psychotherapies) to help these patients cope with their difficulties.

## Author Contributions

JJ and AU contributed to the conception and the design of the study. Data were screened by JJ and CV. JJ, AJ, and CE assessed the material. JJ extracted the data. JJ, CV, and AU analyzed the data. JJ wrote the manuscript. CV rewrote some sections of the manuscript. All authors contributed to manuscript revision and read and approved the submitted version.

## Conflict of Interest

The authors declare that the research was conducted in the absence of any commercial or financial relationships that could be construed as a potential conflict of interest.
