# Supplementary material for: What Do We Know About Young Adult Cardiac Patients' Experience? A Systematic Review
Source: Front Psychol. 2020 Jul 7;11:1119. doi: 10.3389/fpsyg.2020.01119 (PMC7358619; doi:10.3389/fpsyg.2020.01119)
Supplement: Supplementary file 1 [file Table_1.DOCX]

Supplementary Material

# Supplementary Data

| **Box 1.** ScienceDirect search terms  *Each line corresponds to a different search*  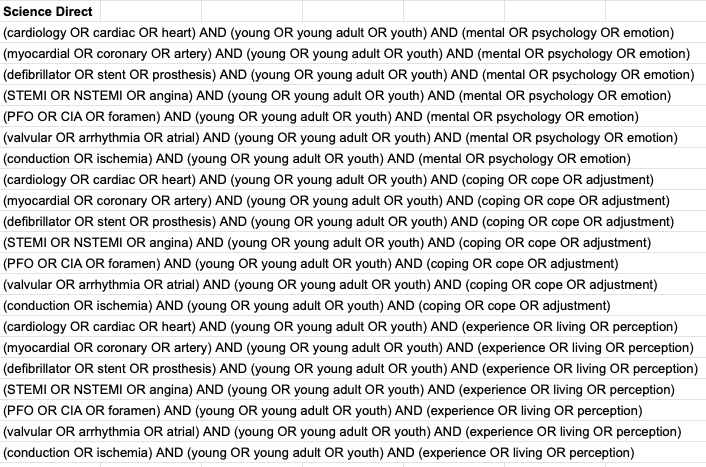 |
| --- |

| **Box 2.** Other database search terms  **PsycINFO equation** (Words in italics are in French.)  **(abstract)**  cardio* OR cardia* OR myocard* OR heart OR *cœur* OR corona* OR defibrillat* OR stent OR prosthesis OR arter* OR ischemi* OR angina OR STEMI OR NSTEMI OR PFO OR CIA OR foramen OR valvula* OR arrhythmia OR atrial OR conduction  **AND (abstract)**  young OR young adult* OR youth OR *adulte* OR *jeune adulte*  **AND (abstract)**  psy* OR coping OR cope OR emotion* OR experience OR adjustment OR *ajustement* OR mental OR perception OR living  **NOT (title)**  stroke OR *avc* OR brain OR cerebral OR *cerveau*  **Pubmed equation**  ((((cardio*[Title/Abstract] OR cardia*[Title/Abstract] OR myocard*[Title/Abstract] OR heart[Title/Abstract] OR coeur[Title/Abstract] OR corona*[Title/Abstract] OR defibrillat*[Title/Abstract] OR stent[Title/Abstract] OR prosthesis[Title/Abstract] OR arter*[Title/Abstract] OR ischemi*[Title/Abstract] OR angina[Title/Abstract] OR STEMI[Title/Abstract] OR NSTEMI[Title/Abstract] OR PFO[Title/Abstract] OR CIA[Title/Abstract] OR foramen[Title/Abstract] OR valvula*[Title/Abstract] OR arrhythmia[Title/Abstract] OR atrial[Title/Abstract] OR conduction[Title/Abstract])) AND (young[Title/Abstract] OR young adult*[Title/Abstract] OR youth[Title/Abstract] OR jeune[Title/Abstract] OR jeune adulte[Title/Abstract])) AND (psy*[Title/Abstract] OR coping[Title/Abstract] OR cope[Title/Abstract] OR emotion*[Title/Abstract] OR experience[Title/Abstract] OR adjustment[Title/Abstract] OR ajustement[Title/Abstract] OR mental[Title/Abstract] OR perception[Title/Abstract] OR living[Title/Abstract])) NOT (stroke[Title] OR avc[Title] OR brain[Title] OR cerebral[Title] OR cerveau[Title])  **Cochrane equation**  (cardio* OR cardia* OR myocard* OR heart OR coeur OR corona* OR defibrillat* OR stent OR prosthesis OR arter* OR ischemi* OR angina OR STEMI OR NSTEMI OR PFO OR CIA OR foramen OR valvula* OR arrhythmia OR atrial OR conduction) AND (young OR young adult* OR jeune OR jeune adulte OR youth) AND (psy* OR coping OR cope OR emotion* OR experience OR adjustment OR mental OR perception OR living) NOT (stroke OR avc OR brain OR cerebral OR cerveau) |
| --- |
